# Catalytic Reactivity Assessment of AgM and CuM (M = Cr, Fe) Catalysts for Dry Reforming of Methane Process with CO_2_

**DOI:** 10.3390/molecules29194597

**Published:** 2024-09-27

**Authors:** Amel Barr, Rafik Benrabaa, Hayat Henni, Laaldja Meddour-Boukhobza, Pascal Roussel, Axel Löfberg

**Affiliations:** 1 Laboratory of Materials Chemistry, Catalysis, and Environment (LCMCE), Department of Physics and Chemistry of Inorganic Materials Faculty of Chemistry, USTHB, El-Alia BP 32, Bab Ezzouar, Algiers 16111, Algeria; barr.amel@hotmail.com (A.B.); boukhobza_meddour@yahoo.fr (L.M.-B.); 2 Laboratory of Physical Chemistry of Materials, Faculty of Sciences and Technology, Chadli Bendjedid University, El-Tarf BP 73, El-Tarf 36000, Algeria; 3 Algerian Petroleum Institute, Avenue 1er Novembre, Boumerdes 35000, Algeria; hayat_h35@yahoo.fr; 4 Univ. Lille, CNRS, Centrale Lille, Univ. Artois, UMR 8181—UCCS—Unit of Catalysis an Solid Chemistry, F-59000 Lille, France; pascal.roussel@univ-lille.fr

**Keywords:** dry reforming of methane, CO_2_ conversion, CuM and AgM catalysts, characterizations

## Abstract

CuM and AgM (M = Cr, Fe) catalysts were synthesized, characterized, and evaluated in methane reforming with CO_2_ with and without pretreatment under a H_2_ atmosphere. Their textural and structural characteristics were evaluated using various physicochemical methods, including XRD, B.E.T., SEM-EDS, XPS, and H_2_-TPR. It was shown that the nature of the species has a significant effect on these structural, textural, and reactivity properties. AgCr catalysts, presenting several oxidation states (Ag^0^, Ag^+1^, Cr^3+^, and Cr^6+^ in Ag, AgCrO_2_, and AgCr_2_O_4_), showed the most interesting catalytic performance in their composition. The intermediate Cr_2_O_3_ phase, formed during the catalytic reaction, played an important role as a catalytic precursor in the in situ production of highly dispersed nanoparticles, being less prone to coke formation in spite of the severe reaction conditions. In contrast, the AgFe catalyst showed low activity and a low selectivity for DRM in the explored temperature range, due to a significant contribution of the reverse water–gas shift reaction, which accounted for the low H_2_/CO ratios.

## 1. Introduction

Natural gas is becoming a significant energy source [1] which can be converted into more valuable compounds through the intermediate of “syngas” or synthesis gas, which is a mixture of carbon monoxide and hydrogen obtained by methane reforming [2,3]. This process aims at converting CH_4_ using an oxidant (such as steam, oxygen, carbon dioxide, or their combination) into syngas [4]. The traditionally used oxidants in methane reforming include steam and oxygen in steam methane reforming (SMR) and partial oxidation reforming (POX), respectively, or the combination of steam and oxygen in autothermal reforming (ATR) [5]. Due to the high CO_2_ footprint of these reference technologies, considerable attention in the literature has shifted towards the dry reforming of methane (DRM), which uses CO_2_ as an oxidant. Thus, DRM is a catalytic process that utilizes both CO_2_ and CH_4_ to convert greenhouse gases into syngas [6,7,8,9,10,11].

SRM:

CH_4_ + H_2_O → CO + 3H_2_ ΔH° = +206 kJ.mol^−1^ (1)

POX:

CH_4_ + 1/2O_2_ → CO + 2H_2_ ΔH° = −36 kJ.mol^−1^ (2)

DRM:

CH_4_ + CO_2_ → 2CO + 2H_2_ ΔH° = +247 kJ.mol^−1^ (3)

Despite the advantages offered by DRM in natural gas conversion and CO_2_ transformation, process challenges persist. The DRM reaction is highly endothermic, and reforming reactions are known to occur near thermodynamic equilibrium conditions. According to previous thermodynamic assessments, a temperature of at least 800 °C or higher is recommended to maintain a high syngas production while mitigating the impacts of competing reactions leading to carbon formation [12,13,14]. Indeed, the dry methane reforming reaction (Equation (3)) can be associated with numerous undesirable reactions, resulting, among others, in carbon deposition (Equations (4)–(6)) or substrates favoring its formation (Equations (7) and (8)).

2CO ⇄ C_(S)_ + H_2_O Boudouard reaction        (4)

CO + H_2_ ⇄ C_(S)_ + H_2_O CO reduction         (5)

CH_4_ ⇄ C_(S)_ + 2H_2_ Pyrolysis of methane        (6)

CO_2_ + H_2_ ⇄ CO + H_2_O Reverse water-gas shift reaction (7)

CH_4_ + H_2_O ⇄ CO + 3H_2_ Steam reforming of methane  (8)

Carbon formation is a major challenge, as it leads to a decreased catalyst efficiency due to rapid deactivation [15], hindering the industrial application of DRM. Previous studies have addressed these challenges by using various approaches related to catalytic materials, reactor design, and energy systems [16,17,18,19,20].

Noble metals (Pt, Rh, Ru, and Ir) demonstrate a superior catalytic efficiency in reforming reactions, particularly in the DRM process. Despite requiring comparatively small amounts of these precious metals [21,22,23,24,25,26,27], given the costly nature and limited availability of noble metals, endeavors have been undertaken to substitute them with more economical d-block metals. Nickel catalysts are commonly employed in the DRM process due to their affordability, wide availability, and catalytic effectiveness comparable to that of noble metals [28,29]. To further enhance DRM catalysts considering carbon formation prevention and additional catalytic activity improvement, using bimetallic Ni catalysts by doping Ni with a second transition metal such as iron (Fe) or copper (Cu) has been suggested. Regarding the latter, research is mainly focused on studying bimetallic catalysts containing Cu [30,31,32,33,34,35,36,37] or copper incorporated into mixed oxide structures [38]. Misture et al. [39] reported increased activity for the DRM reaction with a supported Ni-Cu bimetallic catalyst, with stable conversion over 12 h of operation, without catalytic degradation due to coking. These results were also observed by Reshetenko et al. [40], citing a modified morphology of the catalytic surface due to Cu addition to the Ni-Ni lattice. Lee et al. [41] investigated Cu addition to Ni/Al_2_O_3_ catalysts and found that low Cu loadings helped to decrease the alloying effect caused by Cu enrichment, contributing to reducing carbon formation on the catalysts. However, there is a lack of research on the catalytic activity of catalysts containing only copper as an active metal for such applications [42]. Other studies have investigated bimetallic exsolution for DRM-synthesized Co–Fe and Fe–Ni alloys. The reported CO_2_ conversion values using exsolved alloys (Co–Fe and Fe–Ni) indicated a superior DMR performance of the former [43,44]. Apart from the work of Papargyriou et al. [43], several authors have also explored Fe–Ni alloy exsolution for DRM, particularly for solid oxide fuel cells and electrolyzers [45,46,47,48].

Silver particles hold significant importance due to their exceptional optical, electrical, thermal, and biological attributes. These materials have been suggested for diverse applications across fields like bio-sensors, diagnostics, imaging, catalysts, solar cells, and antibacterial agents [49,50]. As documented in various references, silver has been identified to possess advantageous characteristics in deterring coke formation during reforming reactions [51,52,53,54]. Catalysts based on silver and chromium were synthesized and tested for their hydrogen peroxide decomposition activity with a series of nano-crystalline Ag-Cr-O catalysts. The H_2_O_2_ decomposition activity of the various Ag-Cr-O catalysts showed a continuous decrease with an increasing calcination temperature. It was suggested that the calcination temperature did not alter the proposed reaction mechanism; instead, it could influence the formed phases, which, in turn, generated surface-active Cr^3+^/Cr^6+^ and Cr^3+^/Ag^+^ redox couples involved in the catalytic process [55]. Within the spectrum of chromium oxides, Cr_2_O_3_ stands out as the sole compound retaining thermal stability beyond 500 °C [56,57,58]. Functioning as a catalyst, Cr_2_O_3_ demonstrates a commendable efficacy in both methane combustion [56,57,58,59,60] and ammonia decomposition [61]. Henni et al. [62] found that a Ni/Ag catalyst with a 1:1 ratio exhibited a superior catalytic activity for the dry reforming of methane, achieving 38% CH_4_ and 45% CO_2_ conversion at 650 °C with a H_2_/CO ratio of 0.7 and 71% H_2_ selectivity. The presence of Ag species seemed to enhance the stability and performance of the Ni catalyst in this reaction.

In the current investigation, we prepared a series of catalysts based on copper and silver in conjunction with iron and chromium, denoted as CuM and AgM with (M = Fe, Cr) catalysts, using the coprecipitation method. To characterize these catalysts, we employed various physicochemical techniques, including X-ray diffraction (XRD) and Rietveld refinement, the BET method, X-ray photoelectron spectroscopy (XPS), a scanning electron microscope (SEM) coupled with energy-dispersive X-ray microanalysis (EDX), and temperature-programmed reduction (TPR). We then assessed the activity of these catalysts for hydrogen production through the dry reforming of methane.

## 2. Results

### 2.1. Structural Characterization by XRD

**Figure 1:**
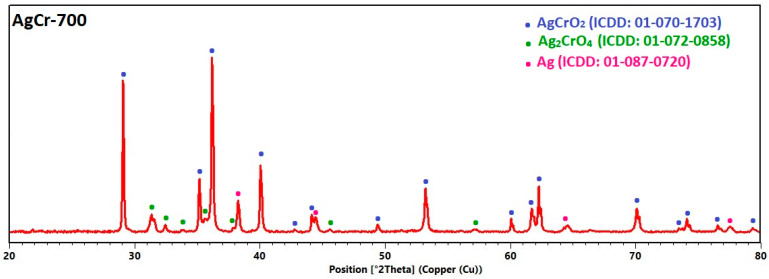

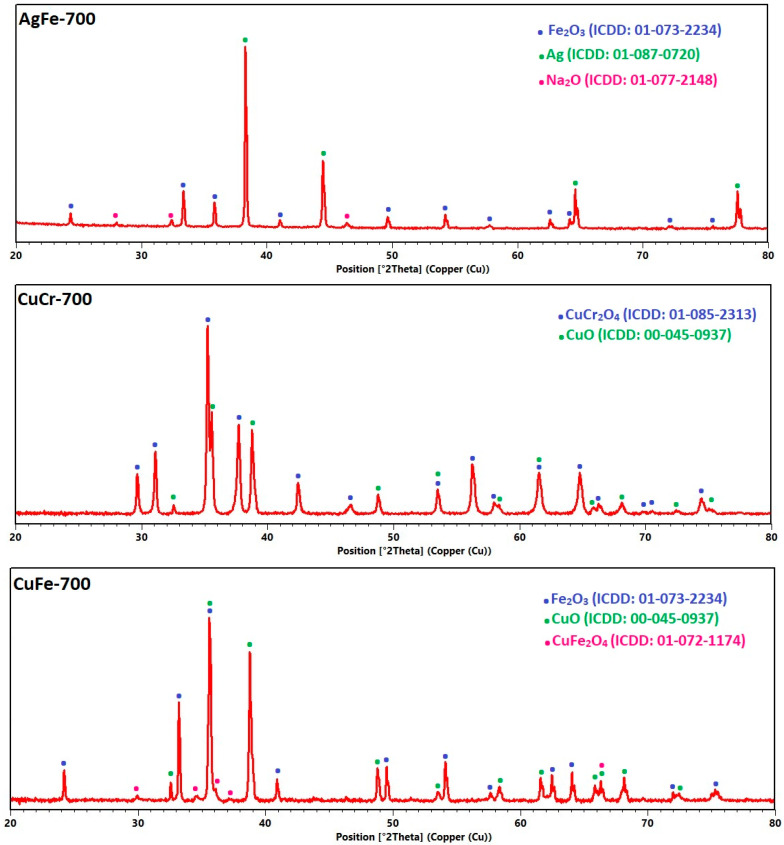
XRD patterns of CuM and AgM (M = Cr or Fe) catalysts prepared by coprecipitation and calcined at 700 °C/7 h.

X-ray diffraction analyses were conducted systematically to investigate the crystal structures and phases of the AgCr-700, AgFe-700, CuCr-700, and CuFe-700 samples. Figure 1 displays the diffraction patterns of each sample, along with the identification of the different crystallographic phases present. For all synthesized formulations, a phase mixture (binary or ternary) was observed. For the AgM (M = Cr, Fe) catalysts, both materials presented a ternary mixture. The AgCr-700 sample, showed the presence of (i) a AgCrO_2_ (ICDD: 01-070-1703) delafossite-type structure, (ii) a Ag_2_CrO_4_ (ICDD: 01-072-0858) spinel-type oxide, and (iii) metallic silver (ICDD: 01-087-0720). The iron-based system of AgFe-700 was mainly composed of the two following phases: (i) hematite-structured Fe_2_O_3_ (ICDD: 01-073-2234) and (ii) metallic silver (ICDD: 01-087-0720), plus some Na_2_O (ICDD: 01-077-2148). The quantitative analysis of these X-ray diffraction (XRD) patterns using the Rietveld method revealed the presence of metallic silver in both the AgCr-700 and AgFe-700 catalysts, but also a significant difference in the formed oxides, despite identical synthesis conditions. The AgCr-700 system was characterized by a more complex composition, comprising two mixed oxides, AgCrO_2_ and Ag_2_CrO_4_, present at percentages of 73% and 14%, respectively, along with metallic silver Ag at 13%. In contrast, the AgFe-700 sample exhibited a single metallic oxide, Fe_2_O_3_, as well as metallic silver, with respective percentages of 55% and 43%. Na_2_O, present as a minor impurity at 2%, originated from the NaOH used during coprecipitation. Its formation during calcination may be attributed to insufficient washing before the process.

For the CuM (M = Cr, Fe) catalysts, a binary system containing copper oxide CuO (ICDD: 00-045-0937) and spinel-phase CuCr_2_O_4_ (ICDD: 01-085-2313) was detected for the chromium-based catalyst, while a ternary mixture composed of CuO (ICDD: 00-045-0937), hematite-structured Fe_2_O_3_ (ICDD: 01-073-2234), and spinel-phase CuFe_2_O_4_ (ICDD: 01-072-1174) was observed for the iron composition. The results of the quantitative Rietveld refinements were the following: the CuCr-700 system showed two different phases of CuO and CuCr_2_O_4_, present at percentages of 27% and 73%, respectively, while the CuFe-700 sample exhibited three distinct phases of CuO, Fe_2_O_3_, and CuFe_2_O_4_ with respective percentages of 52%, 39%, and 9%.

The crystallite size (Cs), calculated using the fundamental parameters approach and presented in Table 1, varied among the samples depending on their structural composition.

### 2.2. Textural and Surface Characterization by BET, SEM-EDX, and XPS

**Figure 2:**
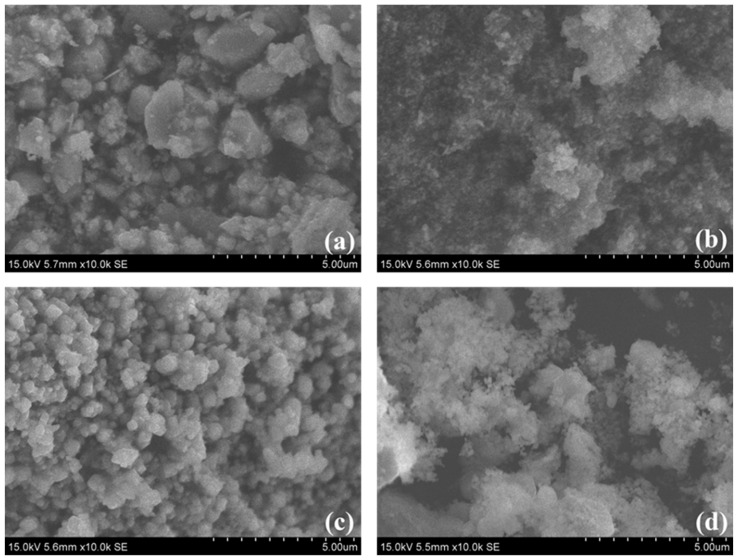
SEM images of (**a**) AgCr-700, (**b**) AgFe-700, (**c**) CuCr-700, and (**d**) CuFe-700.

The specific surface area of the synthetized catalysts did not depend on the nature of the materials (Table 1), as they all exhibited very low specific surface area values not exceeding 6 m^2^/g. This low specific surface area can be explained by the growth of nanoparticles during calcination at 700 °C during 7 h. The calcination temperature significantly affects the surface area of materials. Higher temperatures typically lead to a reduction in surface area due to sintering effects. For instance, increasing the calcination temperature from 400 to 900 °C for Cu-Cr catalysts can reduce their surface area to as low as 10–17 m^2^/g. [63]. The formation of aggregates can also significantly decrease the number of pores. The SEM images in Figure 2 present a series of granular surfaces with textures ranging from irregular to uniform, highlighting how the precursors affected the observed morphology, even when the synthesis conditions were similar. Micrographs of AgFe-700 and CuFe-700 reveal finer, agglomerated particles, while those of AgCr-700 and CuCr-700 show an increasingly uniform and homogeneous distribution of particles with larger sizes. As indicated by the XRD results, the presence of chromium in the AgCr-700 and CuCr-700 catalysts promoted the formation of binary oxides, resulting in more homogeneous morphologies with larger particle sizes and a more uniform distribution of elements. This increased particle size reduced the risk of agglomeration and enhanced homogeneity. In contrast, the iron in the AgFe-700 and CuFe-700 catalysts primarily promoted the formation of simple oxides (with the exception of CuFe_2_O_4_ at 9% in CuFe-700), leading to finer particles. The absence of mixed oxides, combined with the fine structure of the iron-based catalysts, led to particle aggregation, resulting in less homogeneous morphologies. This dissimilarity in the texture of the solids is a potentially critical element for their use as catalysts. 

**Figure 3:**
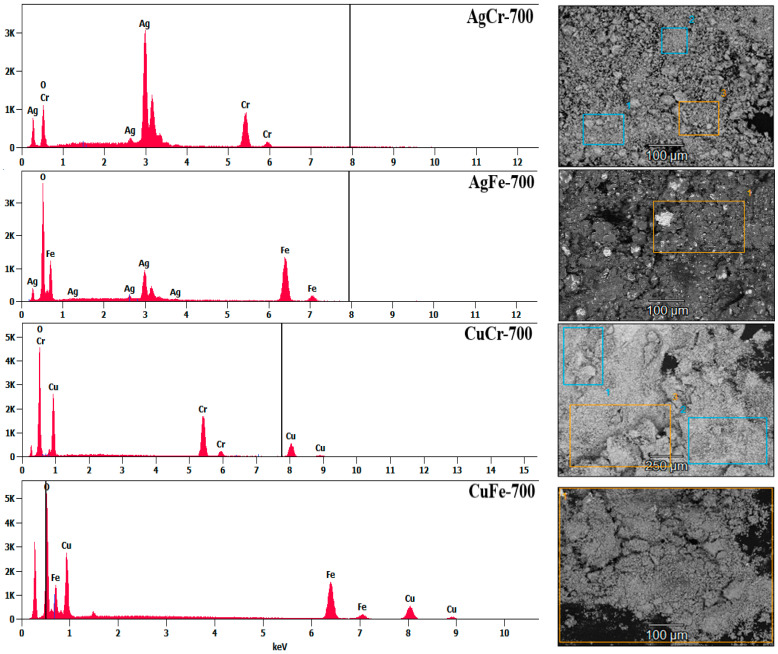
EDS spectra of CuM and AgM (M = Cr or Fe) catalysts prepared by coprecipitation and calcined at 700 °C/7 h.

The elemental composition of the Cu(M) and Ag(M) catalysts was determined through EDS analysis. EDS spectra are presented in Figure 3. The recorded values indicated only the presence of characteristic peaks of the elements Ag, Cu, O, and M (M = Fe, Cr). The atomic ratios of Ag/M and Cu/M (Table 2) were calculated for the different catalysts, showing that the Ag/Cr, Cu/Cr, and Cu/Fe ratios were close to 1, reflecting the uniform phase distribution in these catalysts. However, the Ag/Fe ratio of 0.2 (Table 2) can be attributed to the two following possible phenomena: either the Ag^+^ ions did not fully precipitate during synthesis and remained in solution, or the agglomeration of particles led to an uneven distribution of elements at the microscopic scale.

The chemical state and surface compositions of the catalysts were examined by XPS. The obtained data are summarized in Table 2. The atomic ratios of Ag/Fe and Cu/Cr were close to those obtained by EDS analysis (Table 2), suggesting that the chemical composition in the bulk was close to that on the surface of these catalysts. An enrichment in iron was observed at the surfaces of the AgFe-700 and CuFe-700 catalysts. In addition, an enrichment in Ag was observed for the AgCr-700 catalyst (Ag/Cr = 1.7 against Ag/Cr = 1). 

**Figure 4:**
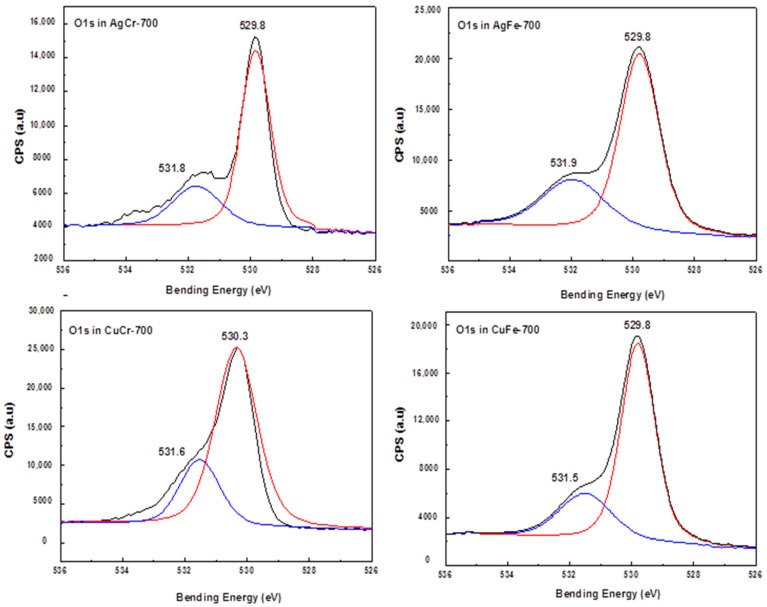
XPS spectra of O 1s species in AgM and CuM (M = Cr or Fe) catalysts prepared by coprecipitation and calcined at 700 °C/7 h.

For all formulations, the photopeak 1s of oxygen (Figure 4) revealed two main components. The first component, corresponding to the lowest binding energy (~530 eV), was related to the lattice oxygen O^2−^, and the second component of a higher binding energy (~531.7 eV) was correlated with the presence of oxygen localized on the outer layer of the solid and belonging to -OH groups or probably to H_2_O adsorbed on the surface.

**Figure 5:**
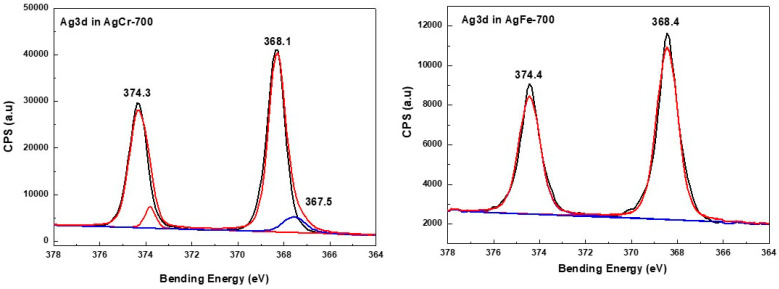
XPS spectra of Ag species in AgM (M = Cr or Fe) catalysts prepared by coprecipitation and calcined at 700 °C/7 h.

**Figure 6:**
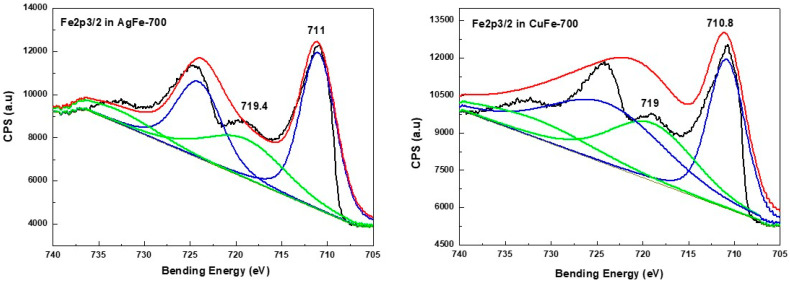
XPS spectra of Fe2p3/2 species in AgM and CuM (M = Cr or Fe) catalysts prepared by coprecipitation and calcined at 700 °C/7 h.

**Figure 7:**
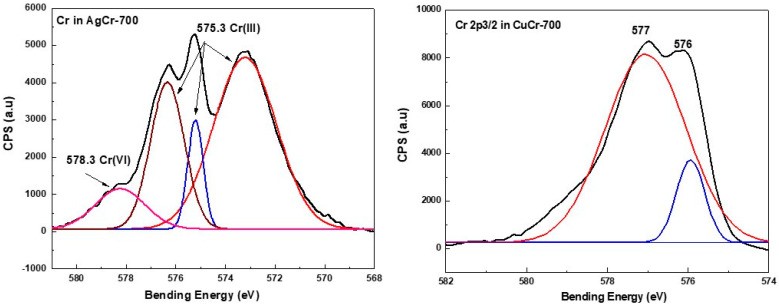
XPS spectra of Cr species in AgM and CuM (M = Cr or Fe) catalysts prepared by coprecipitation and calcined at 700 °C/7 h.

**Figure 8:**
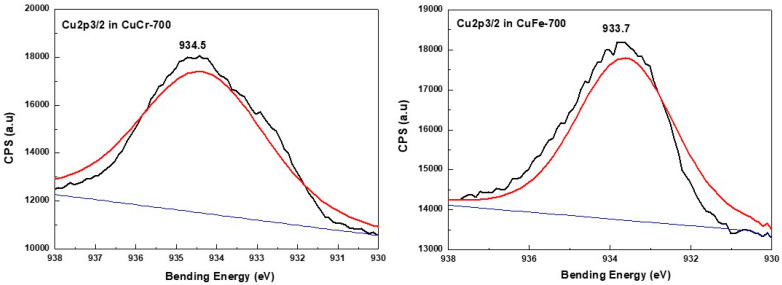
XPS spectra of Cu species in CuM (M = Cr or Fe) catalysts prepared by coprecipitation and calcined at 700 °C/7 h.

In the case of the AgCr-700 system, the Ag 3d5/2 and Ag 3d3/2 core level binding energies located at ~368.1 and ~374.3 eV, respectively, were in good agreement with bulk silver metallic values observed in the literature [64] and our previous work [62]. The decomposition of the corresponding spectrum (Figure 5) showed a component at ~367.5 eV assigned to silver in the Ag^+^ oxidation state. However, this decomposition did not show any other components (Figure 5) for the AgFe-700 sample, explaining that Ag species are only in the metallic oxidation state for this formulation, as suggested by the XRD analysis. The Fe2p3/2 spectra (Figure 6) display the same peak shapes for both the AgFe-700 and CuFe-700 formulations, with corresponding binding energies of 711 eV and 710.8 eV for AgFe-700 and CuFe-700, respectively, characteristic of the presence of Fe (III) species in AgFe and CuFe mixed oxides. In addition, a component appeared at ~719 eV (Figure 6), which is characteristic of the presence of Fe (III) from hematite-structured Fe_2_O_3_ (α or γ) [65,66]. It is very difficult to distinguish between α-Fe_2_O_3_ or γ-Fe_2_O_3_ oxides, since the corresponding binding energies for both oxides are practically the same. The AgCr-700 and CuCr-700 systems showed different Cr2p spectra (Figure 7), with Cr2p line binding energy values of 575.3 and 577 eV for AgCr-700 and CuCr-700, respectively. These values are an excellent indication of the presence of Cr^3+^ in our formulations. In contrast, after the decomposition of the corresponding spectrum for the AgCr-700 system, we note the appearance of a band around 578.3 eV, which can be associated with Cr^6+^ species [65]. The presence of Cr^6+^ species at the surface of the AgCr-700 catalyst was perfectly correlated with the Ag_2_CrO_4_ structure detected by the XRD analysis. Figure 8 shows the Cu 2p3/2 XPS spectra of the CuM (M = Cr or Fe) catalysts. Both the CuCr-700 and CuFe-700 catalysts exhibited similar spectra (934.5 eV for CuCr-700 and 933.7 eV for CuFe-700) and indicated that the copper was only in the (II+) oxidation state. The low and high binding energy peaks were generally attributed to Cu^2+^ located in octahedral and tetrahedral sites of CuCr_2_O_4_ and CuFe_2_O_4_ spineoxides, respectively [67]. Nevertheless and as mentioned by Z. Xiao et al. [67], the peaks were ascribed to Cu^2+^ in CuO, CuCr_2_O_4_, and CuFe_2_O_4_ for the Cu 2p3/2 spectra of both the CuCr-700 and CuFe-700 catalysts.

### 2.3. Reducibility Properties by H_2_-TPR

**Figure 9:**
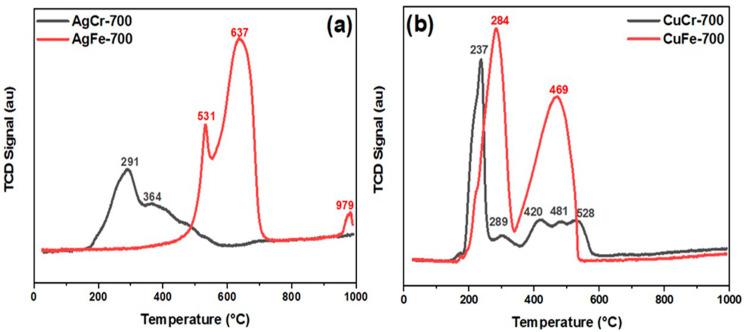
H_2_-TPR profiles of (**b**) AgM (M = Cr or Fe) and (**a**) CuM catalysts prepared by coprecipitation and calcined at 700 °C/7 h.

Figure 9 illustrates the H_2_-TPR profiles of the catalysts. Hydrogen consumption was influenced by both the trivalent cation M (M = Cr or Fe) and the associated metals Cu and Ag. In terms of the influence of the trivalent cation, catalysts containing iron exhibited a higher hydrogen consumption compared to those containing chromium, and when comparing consumption relative to the associated metals (Cu or Ag), the catalysts with Cu showed higher quantities than those with Ag. The H_2_ consumption amounts for each catalyst were the following: 3.2 mmol/g for AgCr-700, 7.9 mmol/g for AgFe-700, 6.3 mmol/g for CuCr-700, and 15.2 mmol/g for CuFe-700. These findings suggest that the AgCr-700 catalyst is more readily reducible, while the CuFe-700 catalyst is less readily reducible. 

Upon closely examining each TPR profile, for the Cu-M catalysts, it is noted that, for the CuCr-700 catalyst (composed of CuO and CuCr_2_O_4_), a prominent peak emerged around 237 °C, accompanied by a much weaker peak around 298 °C. These two peaks could indicate the reduction of CuO to metallic Cu, a process typically occurring between 200 °C and 300 °C, as mentioned in the literature [68]. Additional peaks were also observed at 420 °C, 481 °C, and 528 °C. The peak at 420 °C can be attributed to the reduction of Cu^2+^ in CuCr_2_O_4_. This reduction process is challenging due to limitations in penetration within these particles rather than surface conditions [69], while the peaks at 481 °C and 525 °C can be associated with the reduction of strongly interacting CuO/Cr_2_O_3_ species [70]. For the CuFe-700 catalyst (consisting of CuO, Fe_2_O_3_, and CuFe_2_O_4_), two prominent peaks were detected. As mentioned for the CuCr-700 catalyst, for the peak around 284 °C, even if the reduction peak moved to a higher temperature and the peak height of the reduction peaks also increased, this suggests the reduction of CuO to metallic Cu. Additionally, it is plausible that this peak indicated the partial reduction of CuFe_2_O_4_. The subsequent peak at 469 °C might be linked to the reduction of Fe_2_O_3_ to Fe_3_O_4_. According to the literature, the temperature range of 300–500 °C is associated with the reduction of Fe^3+^ to Fe^2+^ [71,72].

For the H_2_-TPR profiles of Ag-M, interpreting the results requires considering the presence of metallic silver in both samples. Indeed, metallic silver possesses a high thermal and electrical conductivity, which can lead to a more uniform and rapid reduction of the oxides present in the catalyst, resulting in the appearance of peaks at lower temperatures than those expected in the TPR profile [73]. As observed with the AgCr-700 catalyst (mainly composed of AgCrO_2_, Ag_2_CrO_4_, and metallic silver), a peak appeared at 291 °C, followed by a shoulder at 364 °C. The first peak could correspond to the reduction of Ag_2_CrO_4_ to AgCrO_2_ or metallic silver, which typically occurs at a higher temperature. The shoulder observed at 364 °C could indicate the partial reduction of silver or chromium compounds, the reduction of AgCrO_2_ to metallic silver, or the reduction of CrO_3_ to Cr_2_O_3_. 

The influence of metallic silver can also be observed on the AgFe-700 catalyst (composed of metallic silver and Fe_2_O_3_), which exhibited a sharp peak at 637 °C, preceded by a shoulder at 531 °C. These successive peaks can be associated with the reduction of Fe_2_O_3_ to metallic iron. This reduction process typically occurs in two or three steps at temperatures up to 750 °C [72,74]. It is, therefore, assumed that the presence of metallic silver in the catalyst accelerated this reduction. The TPR profile of AgFe-700 showed a final peak at 979 °C of low intensity, which could be related to the interaction between metallic silver and iron oxide and/or the reduction of Fe_2_O_3_ particles located within the bulk of the catalyst, which are not easily reducible at lower temperatures.

### 2.4. Catalytic Properties

**Figure 10:**
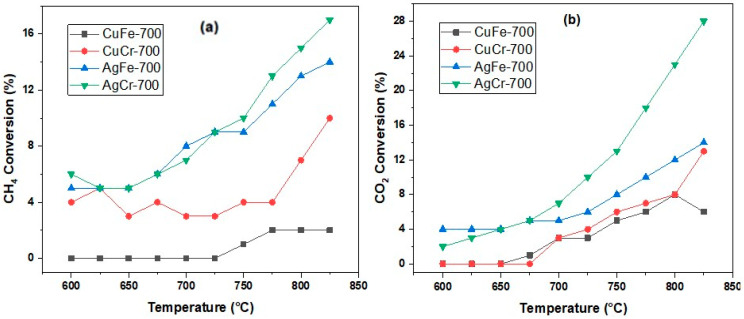
CH_4_ (**a**) and CO_2_ (**b**) conversion obtained on AgM and CuM (M = Cr or Fe) catalysts calcinated at 700 °C (CH_4_ = 20%; CO_2_ = 20%; 100 mg; F = 100 mL/min).

**Figure 11:**
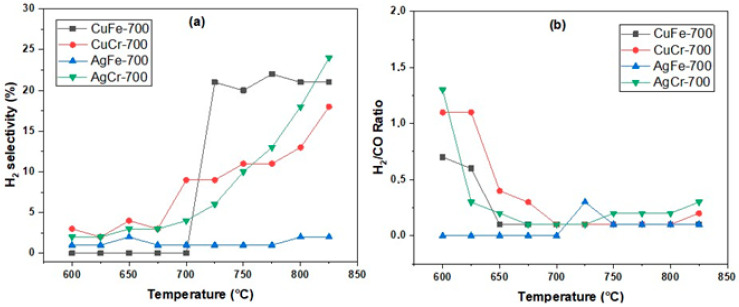
H_2_ selectivity (**a**) and H_2_/CO ratios (**b**) obtained AgM and CuM (M = Cr or Fe) catalysts calcinated at 700 °C (CH_4_ = 20%; CO_2_ = 20%; 100 mg; F = 100 mL/min).

The dry reforming of methane (DRM) was conducted in a temperature range from 600 to 825 °C. The catalytic performances of different formulations were evaluated at each reaction temperature, as shown in Figure 10 and Figure 11, respectively, for the conversions of CH_4_ and CO_2_, as was the selectivity for H_2_ and H_2_/CO ratios. The results revealed that the four catalysts, AgM and CuM, exhibited relatively low conversions for CH_4_ and CO_2_, not exceeding 18% for CH_4_ and 30% for CO_2_. For all formulations, the conversion of CO_2_ exceeded that of CH_4_. The H_2_/CO ratios were very low (below 0.5) compared to the stoichiometry of the reaction (H_2_/CO = 1). In the methane reforming process, several reactions generally occur, including the reverse water–gas shift (RWGS) reaction and the reverse Boudouard reaction, which are favored at high temperatures. The high CO_2_ conversion rates compared to CH_4_, along with the low H_2_/CO ratio, can be attributed to the significant contribution of the RWGS reaction, which could also explain the formation of a substantial amount of water.

The AgCr-700 catalyst demonstrated a high reactant conversion, attributed to the presence of AgCrO_2_ and Ag_2_CrO_4_, which can facilitate the dissociation of CH_4_ and CO_2_. Additionally, the small size of the silver crystallites (26 nm) may have contributed to better dispersion and a higher density of active sites, essential for reactant conversion. Hydrogen selectivity increased progressively with temperature, reaching 24% at 825 °C, suggesting that this catalyst promotes hydrogen formation over a wide temperature range. This behavior may be linked to the thermal stability of the silver and chromium compounds, maintaining their catalytic activity. Regarding the AgFe-700 catalyst, it showed a significant conversion of reactants that increased with temperature, but hydrogen selectivity remained very low, reaching only 2% even at 825 °C. The presence of Fe_2_O_3_ could have favored the reforming reaction, but the low specific surface area limited the number of active sites available for the reaction. Moreover, the presence of metallic silver with a large crystallite size (113 nm) also reduced the active surface area, and the presence of Na_2_O, even in small amounts, could modify the surface properties and impact the overall activity. For the CuCr-700 catalyst, a moderate reactant conversion was observed, increasing with temperature. Hydrogen selectivity followed the same trend, reaching 18% at 825 °C. This performance can be attributed to the predominant presence of CuCr_2_O_4_ with a relatively small crystallite size, improving the dispersion of active sites. As for the CuFe-700 catalyst, the reactant conversion rates remained very low up to a temperature of 650 to 700 °C, where conversion began and increased slightly with temperature. A sudden increase in hydrogen selectivity to 21% from 725 °C may indicate catalytic activation in this temperature range. The interactions between copper and iron oxides became particularly favorable, leading to an improvement in catalytic activity.

The reactivity results of the four catalysts for the dry reforming of methane reaction allow them to be ranked in terms of hydrogen selectivity as follows: AgCr-700, CuFe-700, CuCr-700, and finally, AgFe-700. This ranking shows that the catalytic activity of the AgM and CuM systems depended on both the metal used (Ag or Cu) and the nature of the trivalent cation M (M = Fe or Cr). The AgCr-700 and CuFe-700 catalysts exhibited the highest reactivity. Despite its lower specific surface area, AgCr-700 demonstrated a better reactant conversion. The reduction temperature observed in the H_2_-TPR profiles was lower for AgCr-700 than for CuFe-700 (final peak/shoulder at around 364 °C versus 469 °C), and the Ag/Cr and Cu/Fe ratios (XPS: 1.7 versus 0.3 and EDS: 1.2 versus 0.8) favored an increased dispersion of silver species on the surface of the AgCr-700 catalyst. As obtained in our previous work [75,76,77], the presence of iron generally leads to low activity in the dry reforming of methane, however, the presence of iron in combination with copper yielded more favorable results. Although the CuFe-700 catalyst had a larger specific surface area (6 m^2^/g versus 1 m^2^/g), the AgCr-700 catalyst exhibited a better hydrogen selectivity at 825 °C. This could be attributed to the nature of the silver compounds in the AgCr-700 catalyst, which presented two oxidation states, (0) and (+I), as suggested by the XRD and XPS analyses. These states may be more effective in promoting the reaction towards hydrogen rather than other products.

**Figure 12:**
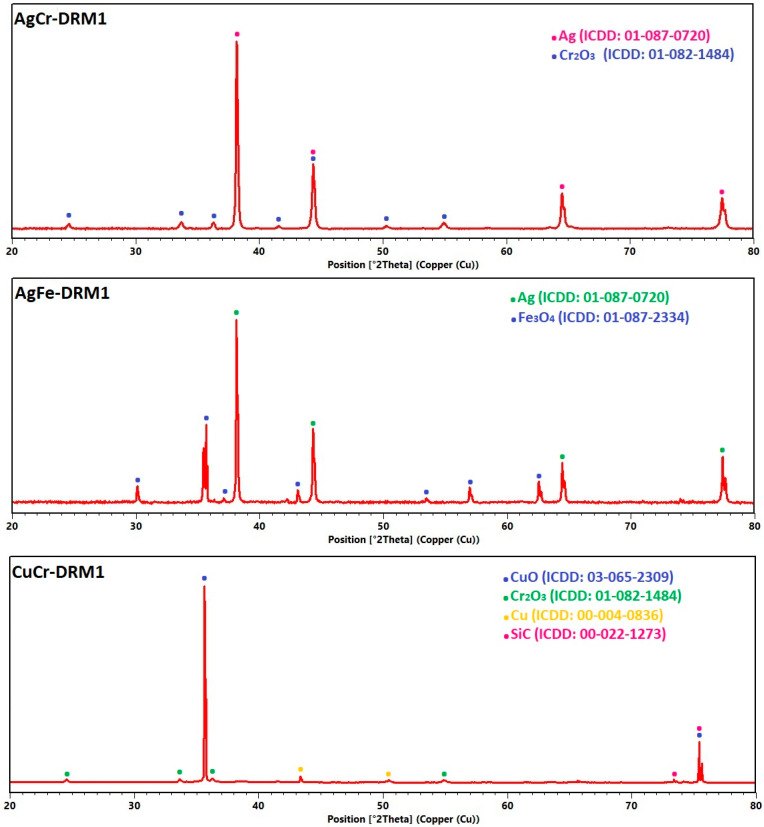

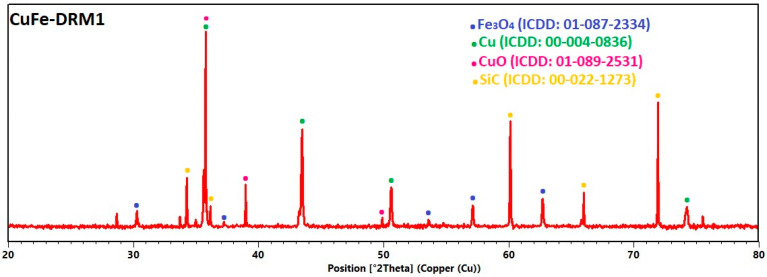
XRD patterns of CuM and AgM (M = Cr or Fe) catalysts after catalytic test for DRM reaction.

To better understand the results of the various catalytic tests, the catalysts were first sieved to remove SiC and then analyzed after the reaction by X-ray diffraction (XRD) to determine the structural changes that occurred (it is worth noting that some SiC peaks may appear in the diffractograms). Figure 12 shows the diffractograms of the four catalysts after the catalytic test (DRM1). The results of the XRD analysis on the CuCr-DRM1 sample revealed significant transformations under the methane dry reforming conditions. Indeed, after the catalytic test, the diffractogram can be indexed using a mixture of CuO, Cu metallic, and Cr_2_O_3_ (plus some peaks coming from the SiC support). The presence of metallic copper and CuO indicated that the catalyst possessed balanced redox properties, facilitating the activation of reactants. These transformations led to the formation of a catalytic system with well-dispersed active phases due to metallic copper, thus explaining a significant catalytic activity compared to other catalysts. The XRD analysis of the CuFe-DRM1 sample also showed structural changes under the methane dry reforming conditions, including the formation of metallic copper and Fe_3_O_4_. Note that while it is possible to explain the diffractogram using either pure copper, it is also possible to conclude the formation of an alloy between Cu and Fe (as, for instance, FeCu_4_, ICDD: 03-065-7002). Metallic copper (or alloy copper and iron), combined with Fe_3_O_4_, constitutes an active catalytic system capable of activating reactants and stabilizing reactivity, even if it does not continuously increase. 

For the AgM catalysts (M = Cr or Fe), the complete reduction of silver species to metallic silver was observed, along with the formation of Cr_2_O_3_ in the AgCr-DRM1 sample and Fe_3_O_4_ in the AgFe-DRM1 sample. The good results obtained with the AgCr-700 catalyst can be attributed to the transformation of the initial phases into Cr_2_O_3_, which is known to be highly active for methane dry reforming, as observed in our previous works [78]. Metallic silver acted as a stabilizer for the catalyst and aided in the dispersion of active phases, and further increasing the temperature enhanced the catalytic activity and selectivity for hydrogen. In contrast, the AgFe-700 catalyst yielded insufficient results for the reaction. Fe_2_O_3_ transformed into Fe_3_O_4_ during the reaction, which is known for its catalytic properties, but the transformation of Fe_2_O_3_ was incomplete. This suggests that the conditions did not favor the formation of even more active phases such as metallic iron or iron carbides. While silver can aid in the dispersion of Fe_2_O_3_, it is not very active for this reaction by itself. Note also that, even in small quantities, the presence of Na_2_O can act as a poison for certain catalysts, reducing their active surface area or altering their redox properties. This could have contributed to the reduced activity of this catalyst.

**Figure 13:**
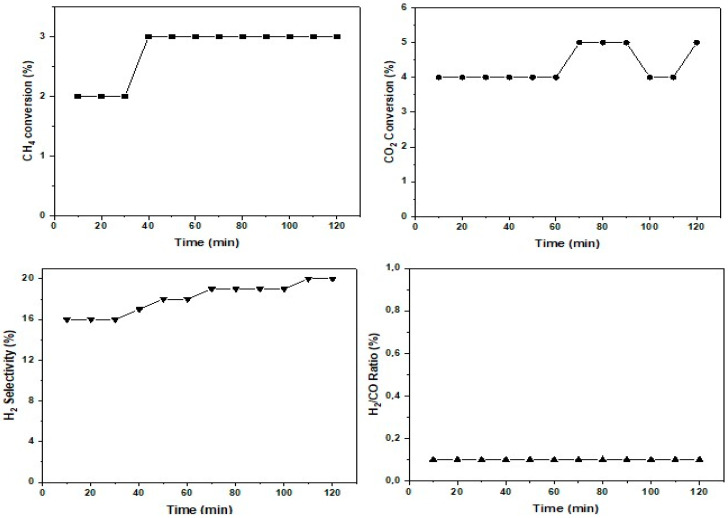
Isothermal test of catalytic performance in term of conversions (CH_4_ and CO_2_), H_2_ selectivity and H_2_/CO ration of AgCr-700 catalyst (CH_4_ = 20%; CO_2_ = 20%; 100 mg; F = 100 mL/min).

In order to more accurately evaluate the catalytic properties of the AgCr-700 catalyst, a new sample was heated from room temperature up to 700 °C under inert gas and then exposed to the same DRM reaction conditions as before, with a reaction time of 2 h. Figure 13 shows the evolution of the CH_4_ and CO_2_ conversions, H_2_ selectivity, and H_2_/CO ratio over time. The conversions of CH_4_ and CO_2_ were relatively low and somewhat decreased compared to those recorded during the previous temperature ramp-up of the DRM. It is possible that certain components of the catalyst started to agglomerate or undergo structural changes, thereby reducing the number of available active sites for the CH_4_ and CO_2_ conversions. Nevertheless, the conversions seemed to remain fairly stable over time, particularly for CH_4_. However, it was still observed that CO_2_ conversion was higher than that of CH_4_ conversion. Although the overall conversion of reactants was low, the selectivity for hydrogen increased over time, surpassing the values recorded during the previous temperature ramp-up for the DRM. This could be explained by the effect of a prolonged exposure to temperature on the adsorption of reactants on the catalyst surface. A weaker adsorption of CH_4_ and CO_2_ could reduce competition for active sites, thus promoting a better selectivity for hydrogen.

**Figure 14:**
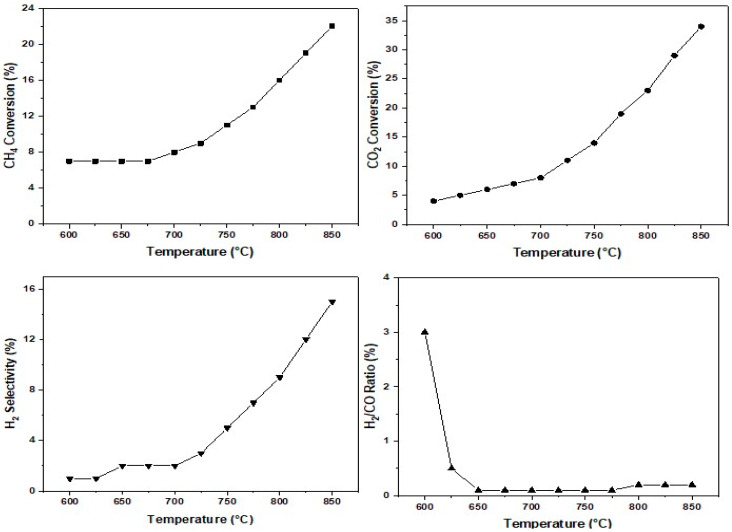
Catalytic performance after reducing H_2_ pretreatment in terms of conversions (CH_4_ and CO_2_), H_2_ selectivity and H_2_/CO ration of AgCr catalyst calcinated at 700 °C (CH_4_ = 20%; CO_2_ = 20%; 100 mg; F = 100 mL/min).

The AgCr-700 catalyst, primarily composed of AgCrO_2_, Ag_2_CrO_4_, and metallic silver, underwent hydrogen pretreatment at 400 °C in a mixture of 10% H_2_ and 90% Ar for one hour. Figure 14 shows the evolution of CH_4_ and CO_2_ conversions, H_2_ selectivity, and H_2_/CO as a function of temperature. This treatment is commonly used to reduce oxidized species and activate catalytic sites. However, following this pretreatment, a decline in catalytic performance was observed as follows: although the conversion rates of CH_4_ and CO_2_ remained within the same range as before the pretreatment, hydrogen selectivity was halved. Several explanations can be offered for this performance decrease. Hydrogen pretreatment may have reduced the silver compounds to a pure metallic state, which is not necessarily the most active state for the reforming reaction. The excessive reduction or modification of active sites could have affected the reactivity and selectivity of the catalyst. Additionally, the pretreatment temperature may have induced changes in the catalyst structure, such as the sintering of metal particles, thereby reducing the specific surface area and the number of available active sites. It is also possible that pretreatment promoted the formation of carbon deposits on the catalyst surface, blocking active sites and reducing hydrogen selectivity. Furthermore, pretreatment may have affected the dispersion of metals on the catalyst, which can also decrease hydrogen selectivity.

**Figure 15:**
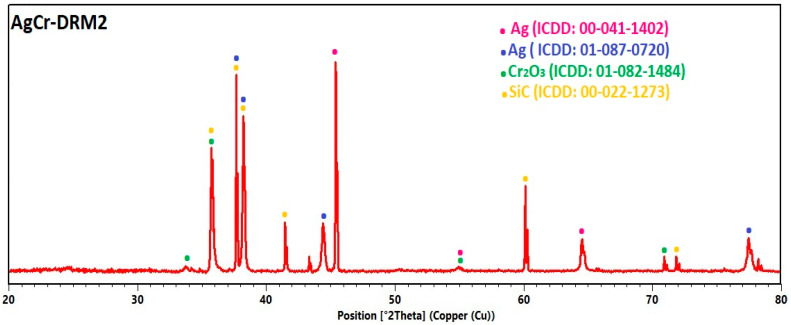
XRD patterns of AgCr-700 catalyst after isothermal test for DRM reaction.

The AgCr-700 catalyst tested in an isothermal reaction was also analyzed by XRD. Figure 15 shows the diffractogram of the sample, with the results, as with the previous four samples, indicating a significant structural change. The silver species were fully reduced to metallic silver, and the extended reaction time led to the appearance of metallic silver in the two following structures: cubic and hexagonal. This last polymorph is not so common, but has already been observed in extreme conditions such as reductive thin films synthesis or nanoparticles [79,80,81]. The reaction time did not affect the reduction of chromium species, as they were reduced to Cr_2_O_3_, similar to the AgCr-DRM1 sample. These results confirm the slight decrease in reactivity observed during the isothermal test due to the excessive reduction of silver species.

## 3. Materials and Methods

### 3.1. Chemicals

Copper (II) nitrate hexahydrate (Cu(NO_3_)_2_.6H_2_O, ≥98%, Sigma Aldrich, Saint Louis, MO, USA), silver (I) nitrate (AgNO_3_, 99%, Sigma Aldrich, Saint Louis, MO, USA), iron (III) nitrate nonahydrate (Fe(NO_3_)_3_.9H_2_O, ≥98%, Sigma Aldrich, Saint Louis, MO, USA), chromium (III) nitrate nonahydrate (Cr(NO_3_)_3_.9H_2_O, ≥99%, Sigma Aldrich, Saint Louis, MO, USA), and sodium hydroxide (NaOH, ≥98%, Sigma Aldrich, Saint Louis, MO, USA) were used in the catalysts’ preparation. All reagents were of analytical grade and were used without any further purification. Distilled water and absolute ethanol were used in the synthesis and washing processes.

### 3.2. Catalyst Preparation

The catalysts were prepared by the coprecipitation method using metallic precursors such as copper, silver, iron, and chromium nitrates. Stoichiometric amounts of nitrates corresponding to the Cu/M = 1:1 and Ag/M = 1:1 molar ratios (M = Cr, Fe) were individually dissolved in distilled water at room temperature. These solutions were then mixed in a single container. The coprecipitation of the metal ions was achieved by adding NaOH solution to adjust the pH to 10, with stirring at room temperature. The resulting precipitate was separated from the solution by centrifugation, followed by washing with distilled water until neutralization (pH = 7) and absolute ethanol to remove impurities. After a final centrifugation, the solid was dried at 80 °C for 12 h. Finally, the dried material was ground in a mortar to obtain a fine and homogeneous powder. The obtained powder underwent a calcination step at a temperature of 700 °C for a duration of 7 h under static air. The obtained catalysts were denoted as AgCr-700, AgFe-700, CuCr-700, and CuFe-700.

### 3.3. Catalysts Characterization

Several physicochemical techniques were employed to characterize the catalysts. Powder X-ray diffraction (PXRD) analysis was carried out using a Bruker AXS D8 Advance diffractometer operating (Bruker AXS GmbH, Karlsruhe, Germany) in a Bragg–Brentano geometry with Cu Kα radiation (λ = 1.5418 Å) and equipped with a LynxEye detector. Data were collected at room temperature with a 0.02° step size and a counting time of 0.5 per step. Phase identification was conducted using the EVA software (version 6.1.0.4, Bruker AXS GmbH, Karlsruhe, Germany). To gain deeper insights into the crystalline structures of the samples, we employed Rietveld refinement to fit the XRD patterns using the structural models outlined in the experimental section. The quantitative analysis involved Rietveld refinement of the XRD data using the Marquardt least-squares algorithm, implemented in the JANA2006 software package. Through Rietveld full-pattern fitting with various models, we determined the unit cell parameters, weight percentages, and crystallite sizes of the individual phases present in each sample (using fundamental parameters, as implemented in the Jana2006 software).

X-ray photoelectron spectroscopy (XPS) analysis was conducted using a Kratos Analytical AXIS Ultra DLD spectrometer (Vacuum Generators, Seoul, Republic of Korea) equipped with a monochromatic Al Kα X-ray source. All XPS binding energies were calibrated with respect to the C1s core level at 285 eV.

A HITACHI SU3800 SEM with a Bruker Quantax ultrathin window EDX detector (S-3400N, Hitachi, Tokyo, Japan) was used for SEM/EDX (scanning electron microscope/energy dispersive X-ray) analyses. The chemical components of the particles were determined from EDX data acquired in region mode within each particle.

The specific surface areas (S_BET_) of the different solids were determined from nitrogen adsorption isotherms measured at −196 °C using (30 vol% N_2_/He) nitrogen adsorption experiments conducted on the FlowSorb III instrument (Norcross, GA, USA). All samples were degassed for 30 min at 150 °C prior to analysis.

The reducibility of the catalysts was analyzed using temperature-programmed reduction (TPR). Hydrogen temperature-programmed reduction (H_2_-TPR) was conducted using a Micromeritics AutoChem II 2920 (Norcross, GA, USA) apparatus equipped with a thermal conductivity detector (TCD) to monitor hydrogen consumption. Following the calibration of hydrogen on the TCD, the samples were enclosed in a U-shaped quartz tube reactor and pretreated under an argon atmosphere to eliminate surface impurities. Subsequently, the temperature was increased from 25 to 1000 °C at a rate of 5 °C/min in a stream containing 5 vol% H_2_/Ar.

### 3.4. Catalytic Reforming Experiments

The catalytic CO_2_ reforming of methane tests were conducted at atmospheric pressure using a fixed-bed U-type quartz reactor. Prior to loading into the reactor, 200 mg of catalyst was thoroughly mixed with SiC powder. The gas mixture, consisting of CH_4_:CO_2_:Ar = 20:20:60 with a total flow rate of 100 mL/min, was introduced and the catalytic reaction was performed in temperature-programmed mode, ramping from room temperature to 825 °C at a heating rate of 5 °C/min. The gas flow was continuously monitored online using a Prisma 200 Pfeiffer mass spectrometer (OmniStar, Pfeiffer Vacuum, Asslar, Germany). Isothermal reactivity was assessed by heating a new catalyst sample to the reaction temperature (700 °C) in argon, followed by exposure to the same reaction conditions for approximately 2 h.

Alternatively, catalyst activation was achieved through 1 h of pretreatment at 400 °C using a gas mixture containing 10 vol% H_2_ and 90% argon Ar to initiate catalyst reduction. The activated catalyst was then utilized for the dry reforming reaction of methane (CH_4_) with carbon dioxide (CO_2_) under the same conditions.

## 4. Conclusions

CuM and AgM catalysts (M = Cr or Fe) were prepared using the coprecipitation method and then calcined at 700 °C. Structural and textural characterizations revealed significant differences depending on the precursors used for each sample. XRD showed that silver-based catalysts revealed the presence of metallic silver, while chromium-based catalysts favored the formation of mixed oxides. BET analysis indicated a low specific surface area for all samples, with values not exceeding 6 m^2^/g. Grain morphology and elemental composition were determined by SEM-EDX, showing finer and more agglomerated particles for the CuFe-700 and AgFe-700 catalysts, while the CuCr-700 and AgCr-700 catalysts exhibited larger particle sizes with a more homogeneous distribution. The Cu/M and Ag/M atomic ratios were close to 1, except for the Ag/Fe ratio. The AgCr-700 catalyst was the most easily reducible, displaying lower reduction temperatures due to the presence of metallic silver in its composition. The CuM and AgM catalysts were tested for the catalytic reaction of dry methane reforming, and the results showed relatively low catalytic activity, especially for the AgFe-700 catalyst. The AgCr-700 catalyst was the most efficient of the four catalysts and, thus, underwent stability testing and pretreatment to improve its catalytic performance. The catalyst showed a good stability throughout the test (2 h), with an increasing reactivity over time. The pretreatment, however, halved the catalytic activity, indicating that this step is not beneficial for this catalyst in the catalytic reaction of the dry reforming of methane.

## Figures and Tables

**Table 1:** Rietveld refinement and specific surface area results of CuM and AgM catalysts.

Catalysts	XRD Data									S.S.A.
	Detected Phases	Cs (nm)	Lattice Parameters						Phase (%)	S_B_._E_._T_. (m^2^/g)
			a (Å)	b (Å)	c (Å)	α (°)	β (°)	γ (°)		
AgCr-700	AgCrO_2_	64 (4)	2.9860 (4)	2.9860 (4)	18.509 (3)	90	90	120	72 (2)	1
	Ag_2_CrO_4_	41 (12)	10.05 (1)	7.028 (7)	5.543 (6)	90	90	90	15 (2)	
	Ag	26 (5)	4.088 (2)	4.088 (2)	4.088 (2)	90	90	90	13 (1)	
AgFe-700	Fe_2_O_3_	89 (3)	5.0360 (2)	5.0360 (2)	13.7530 (8)	90	90	120	55.0 (6)	4
	Ag	113 (2)	4.069 (7)	4.069 (7)	4.069 (7)	90	90	90	43 (2)	
	Na_2_O	59 (9)	5.5550 (7)	5.5550 (7)	5.5550 (7)	90	90	90	2.2 (3)	
CuCr-700	CuO	70 (12)	4.687 (2)	3.426 (2)	5.130 (3)	90	99.52 (3)	90	27 (2)	4
	CuCr_2_O_4_	46 (4)	6.028 (2)	6.028 (2)	7.806 (3)	90	90	90	73 (2)	
CuFe-700	CuO	71 (2)	4.6870 (2)	3.4251 (2)	5.1316 (3)	90	99.497 (3)	90	52.1 (9)	6
	Fe_2_O_3_	79 (2)	5.0366 (2)	5.0366 (2)	13.7505 (8)	90	90	120	39.2 (4)	
	CuFe_2_O_4_	21 (3)	5.819 (3)	5.819 (3)	8.6982 (6)	90	90	90	8.7 (6)	

**Table 2:** EDS and XPS data of CuM and AgM (M = Cr or Fe) catalysts.

Catalysts	EDS Data	XPS Data		
	Ag/M or Cu/M (M = Cr or Fe)	Ag or Cu * (eV)	Cr or Fe * (eV)	Ag/M or Cu/M (M = Cr or Fe)
AgCr-700	1.2	368.1	575.3	1.7
AgFe-700	0.2	368.4	711	0.2
CuCr-700	1	934.5	577	1.2
CuFe-700	0.8	933.7	710.8	0.3

* Bending Energy.

## Data Availability

Data available within the article.

## References

[1] UNECE (2019). How Natural Gas Can Support the Uptake of Renewable Energy.

[2] Burkart M.D., Hazari N., Tway C.L., Zeitler E.L. (2019). Opportunities and Challenges for Catalysis in Carbon Dioxide Utilization. ACS Catal..

[3] Karakaya C., Kee R.J. (2016). Progress in the direct catalytic conversion of methane to fuels and chemicals. Prog. Energy Combust. Science.

[4] Elbashir N.O. (2018). Introduction to natural gas monetization. Natural Gas Processing from Midstream to Downstream.

[5] York A.P.E., Xiao T., Green M.L.H. (2003). Brief overview of the partial oxidation of methane to synthesis gas. Top. Catal..

[6] Challiwala M.S., Ghouri M.M., Linke P., El-Halwagi M.M., Elbashir N.O. (2017). A combined thermo-kinetic analysis of various methane reforming technologies: Comparison with dry reforming. J. CO2 Util..

[7] Fidalgo B., Domínguez A., Pis J.J., Menéndez J.A. (2008). Microwave-assisted dry reforming of methane. Int. J. Hydrogen Energy.

[8] Jang W.-J., Shim J.-O., Kim H.-M., Yoo S.-Y., Roh H.-S. (2019). A review on dry reforming of methane in aspect of catalytic properties. Catal. Today.

[9] Usman M., Daud W.M.A.W., Abbas H.F. (2015). Dry reforming of methane: Influence of process parameters—A review. Renew. Sustain. Energy Rev..

[10] Wolf M., de Oliveira A.L., Taccardi N., Maisel S., Heller M., Antara S.K., Søgaard A., Felfer P., Görling A., Haumann M. (2023). Dry reforming of methane over gallium-based supported catalytically active liquid metal solutions. Commun. Chem..

[11] Patel N., Al A.S., Bamatraf N.A., Osman A.I., Alreshaidan S.B., Fakeeha A.H., Wazeer I., Kumar R. (2024). 5Ni/MgO and 5Ni/MgO + MOx (M = Zr, Ti, Al) Catalyst for Hydrogen Production via Dry Reforming of Methane: Promotor-Free, Cost-Effective, and Handy Catalyst System. Catal. Lett..

[12] Hussien A.G.S., Polychronopoulou K. (2022). A Review on the Different Aspects and Challenges of the Dry Reforming of Methane (DRM) Reaction. Nanomaterials.

[13] He C., Wu S., Wang L., Zhang J. (2022). Recent advances in photo-enhanced dry reforming of methane: A review. J. Photochem. Photobiol. C Photochem. Rev..

[14] Lavoie J.-M. (2014). Review on dry reforming of methane, a potentially more environmentally-friendly approach to the increasing natural gas exploitation. Front. Chem..

[15] Pakhare D., Spivey J. (2014). A review of dry (CO_2_) reforming of methane over noble metal catalysts. Chem. Soc. Rev..

[16] Benguerba Y., Virginie M., Dumas C., Ernst B. (2017). Computational fluid dynamics study of the dry reforming of methane over Ni/Al_2_O_3_ catalyst in a membrane reactor. Coke deposition. Kinet. Catal..

[17] Durán P., Sanz-Martínez A., Soler J., Menéndez M., Herguido J. (2019). Pure hydrogen from biogas: Intensified methane dry reforming in a two-zone fluidized bed reactor using permselective membranes. Chem. Eng. J..

[18] Olah G.A., Goeppert A., Czaun M., Prakash G.K.S. (2013). Bi-reforming of methane from any source with steam and carbon dioxide exclusively to metgas (CO–2H_2_) for methanol and hydrocarbon synthesis. J. Am. Chem. Soc..

[19] Shi L.-Y., Li Y.X., Xue D.M., Tan P., Jiang Y., Liu X.Q., Sun L.B. (2020). Fabrication of highly dispersed nickel in nanoconfined spaces of as-made SBA-15 for dry reforming of methane with carbon dioxide. Chem. Eng. J..

[20] Song Y., Ozdemir E., Ramesh S., Adishev A., Subramanian S., Harale A., Albuali M., Fadhel B.A., Jamal A., Moon D. (2020). Dry reforming of methane by stable Ni–Mo nanocatalysts on single-crystalline MgO. Science.

[21] Androulakis A., Yentekakis I.V., Panagiotopoulou P. (2023). Dry reforming of methane over supported Rh and Ru catalysts: Effect of the support (Al_2_O_3_, TiO_2_, ZrO_2_, YSZ) on the activity and reaction pathway. Int. J. Hydrogen Energy.

[22] Liang D., Wang Y., Chen M., Xie X., Li C., Wang J., Yuan L. (2024). Dry reforming of methane for syngas production over noble metals modified M-Ni@S-1 catalysts (M = Pt, Pd, Ru, Au). Int. J. Hydrogen Energy.

[23] Maina S.C.P., Ballarini A.D., Vilella J.I., de Miguel S.R. (2020). Study of the performance and stability in the dry reforming of methane of doped alumina supported iridium catalysts. Catal. Today.

[24] Özkara-Aydınoğlu Ş., Özensoy E., Aksoylu A.E. (2009). The effect of impregnation strategy on methane dry reforming activity of Ce promoted Pt/ZrO_2_. Int. J. Hydrogen Energy.

[25] Wang H.Y., Ruckenstein E. (2000). Carbon dioxide reforming of methane to synthesis gas over supported rhodium catalysts: The effect of support. Appl. Catal. A Gen..

[26] Ballarini A.D., de Miguel S.R., Jablonski E.L., Scelza O.A., Castro A.A. (2005). Reforming of CH4 with CO2 on Pt-supported catalysts: Effect of the support on the catalytic behaviour. Catal. Today.

[27] Da Fonseca R.O., Rabelo-Neto R.C., Simões R.C.C., Mattos L.V., Noronha F.B. (2020). Pt supported on doped CeO_2_/Al_2_O_3_ as catalyst for dry reforming of methane. Int. J. Hydrogen Energy.

[28] Abdullah B., Ghani N.A.A., Vo D.-V.N. (2017). Recent advances in dry reforming of methane over Ni-based catalysts. J. Clean. Prod..

[29] Wang Y., Yao L., Wang S., Mao D., Hu C. (2018). Low-temperature catalytic CO2 dry reforming of methane on Ni-based catalysts: A review. Fuel Process. Technol..

[30] Faro M.L., Frontera P., Antonucci P., Aricò A.S. (2015). Ni–Cu based catalysts prepared by two different methods and their catalytic activity toward the ATR of methane. Chem. Eng. Res. Des..

[31] Pendem S., Mondal I., Shrotri A., Rao B.S., Lingaiah N., Mondal J. (2018). Unraveling the structural properties and reactivity trends of Cu–Ni bimetallic nanoalloy catalysts for biomass-derived levulinic acid hydrogenation. Sustain. Energy Fuels.

[32] Zhang R., Guo X., Wang B., Ling L. (2015). Insight into the effect of CuNi (111) and FeNi (111) surface structure and second metal composition on surface carbon elimination by O or OH: A comparison study with Ni (111) surface. J. Phys. Chem. C.

[33] Lee J., Bae Y., Hong K., Hong J. (2022). Comparative evaluation of Ni-based bimetallic catalysts for dry reforming of methane at low temperature: The effect of alloy itself on performance. Int. J. Energy Res..

[34] Sagar T.V., Padmakar D., Lingaiah N., Prasad P.S.S. (2019). Influence of solid solution formation on the activity of CeO_2_ supported Ni–Cu mixed oxide catalysts in dry reforming of methane. Catal. Lett..

[35] Song K., Lu M., Xu S., Chen C., Zhan Y., Li D., Au C., Jiang L., Tomishige K. (2018). Effect of alloy composition on catalytic performance and coke-resistance property of Ni-Cu/Mg (Al) O catalysts for dry reforming of methane. Appl. Catal. B Environ..

[36] Rahemi N., Haghighi M., Babaluo A.A., Jafari M.F., Khorram S. (2013). Non-thermal plasma assisted synthesis and physicochemical characterizations of Co and Cu doped Ni/Al_2_O_3_ nanocatalysts used for dry reforming of methane. Int. J. Hydrogen Energy.

[37] Zhang J., Wang H., Dalai A.K. (2007). Development of stable bimetallic catalysts for carbon dioxide reforming of methane. J. Catal..

[38] Sutthiumporn K., Maneerung T., Kathiraser Y., Kawi S. (2012). CO_2_ dry-reforming of methane over La0. 8Sr0. 2Ni0. 8M0. 2O_3_ perovskite (M = Bi, Co, Cr, Cu, Fe): Roles of lattice oxygen on C–H activation and carbon suppression. Int. J. Hydrogen Energy.

[39] Misture S.T., McDevitt K.M., Glass K.C., Edwards D.D., Howe J.Y., Rector K.D., He H., Vogel S.C. (2015). Sulfur-resistant and regenerable Ni/Co spinel-based catalysts for methane dry reforming. Catal. Sci. Technol..

[40] Reshetenko T.V., Avdeeva L.B., Ismagilov Z.R., Chuvilin A.L., Ushakov V.A. (2003). Carbon capacious Ni-Cu-Al_2_O_3_ catalysts for high-temperature methane decomposition. Appl. Catal. A Gen..

[41] Lee J.-H., Lee E.-G., Joo O.-S., Jung K.-D. (2004). Stabilization of Ni/Al_2_O_3_ catalyst by Cu addition for CO_2_ reforming of methane. Appl. Catal. A Gen..

[42] Bian Z., Zhong W., Yu Y., Jiang B., Kawi S. (2020). Cu/SiO_2_ derived from copper phyllosilicate for low-temperature water-gas shift reaction: Role of Cu+ sites. Int. J. Hydrogen Energy.

[43] Papargyriou D., Miller D.N., Irvine J.T.S. (2019). Exsolution of Fe–Ni alloy nanoparticles from (La, Sr)(Cr, Fe, Ni) O_3_ perovskites as potential oxygen transport membrane catalysts for methane reforming. J. Mater. Chem. A.

[44] Joo S., Kwon O., Kim K., Kim S., Kim H., Shin J., Jeong H.Y., Sengodan S., Han J.W., Kim G. (2019). Cation-swapped homogeneous nanoparticles in perovskite oxides for high power density. Nat. Commun..

[45] Du Z., Yi S., Xia Q., Gong Y., Zhang Y., Cheng X., Li Y., Gu L., Świerczek K. (2016). High-performance anode material Sr2FeMo0. 65Ni0. 35O6− δ with in situ exsolved nanoparticle catalyst. ACS Nano.

[46] Wang Y., Liu T., Li M., Xia C., Zhou B., Chen F. (2016). Exsolved Fe–Ni nano-particles from Sr 2 Fe 1.3 Ni 0.2 Mo 0.5 O 6 perovskite oxide as a cathode for solid oxide steam electrolysis cells. J. Mater. Chem. A.

[47] Li J., Yu Y., Yin Y.-M., Zhou N., Ma Z.-F. (2017). A novel high performance composite anode with in situ growth of Fe-Ni alloy nanoparticles for intermediate solid oxide fuel cells. Electrochim. Acta.

[48] Zhu T., Troiani H.E., Mogni L.V., Han M., Barnett S.A. (2018). Ni-substituted Sr (Ti, Fe) O_3_ SOFC anodes: Achieving high performance via metal alloy nanoparticle exsolution. Joule.

[49] Jun B.-H. (2019). Silver nano/microparticles: Modification and applications. Int. J. Mol. Sci..

[50] Pham X.-H., Kim J., Jun B.-H. (2020). Silver nano/microparticles: Modification and applications 2.0. Int. J. Mol. Sci..

[51] Suttikul T., Nuchdang S., Rattanaphra D., Photsathain T., Phalakornkule C. (2022). Plasma-assisted CO_2_ reforming of methane over Ni-based catalysts: Promoting role of Ag and Sn secondary metals. Int. J. Hydrogen Energy.

[52] Yu M., Zhu Y.A., Lu Y., Tong G., Zhu K., Zhou X. (2015). The promoting role of Ag in Ni-CeO_2_ catalyzed CH4-CO_2_ dry reforming reaction. Appl. Catal. B Environ..

[53] Vang R.T., Honkala K., Dahl S., Vestergaard E.K., Schnadt J., Lægsgaard E., Clausen B.S., Nørskov J.K., Besenbacher F. (2005). Controlling the catalytic bond-breaking selectivity of Ni surfaces by step blocking. Nat. Mater..

[54] Parizotto N.V., Rocha K.O., Damyanova S., Passos F.B., Zanchet D., Marques C.M.P., Bueno J.M.C. (2007). Alumina-supported Ni catalysts modified with silver for the steam reforming of methane: Effect of Ag on the control of coke formation. Appl. Catal. A Gen..

[55] Abu-Zied B.M., Ali T.T. (2018). Fabrication, characterization and catalytic activity measurements of nano-crystalline Ag-Cr-O catalysts. Appl. Surf. Sci..

[56] Wang J., Gupta A., Klein T.M. (2008). Plasma enhanced chemical vapor deposition of Cr_2_O_3_ thin films using chromium hexacarbonyl (Cr(CO)6) precursor. Thin Solid Films.

[57] Wang T.G., Jeong D., Liu Y., Wang Q., Iyengar S., Melin S., Kim H.K. (2012). Study on nanocrystalline Cr_2_O_3_ films deposited by arc ion plating: II. Mechanical and tribological properties. Surf. Coat. Technol..

[58] Alharbi W. (2023). Structural, compositional study, and thermal behavior of TiO_2_/Cr_2_O_3_/GO nanocomposite for methylene blue degradation. J. Photochem. Photobiol. A Chem..

[59] Zheng S., Wang S.X., Li S.J. (2021). Influence of Cr_2_O_3_ on Catalytic Performance of MXOY(M = Cu, Ni, Ce)/Al_2_O_3_-ZrO_2_ for Methane Combustion. J. Eng. Sci. Technol. Rev..

[60] Ordóñez S., Paredes J.R., Díez F.V. (2008). Sulphur poisoning of transition metal oxides used as catalysts for methane combustion. Appl. Catal. A Gen..

[61] Li L., Zhu Z.H., Wang S.B., Yao X.D., Yan Z.F. (2009). Chromium oxide catalysts for COx-free hydrogen generation via catalytic ammonia decomposition. J. Mol. Catal. A Chem..

[62] Henni H., Benrabaa R., Roussel P., Löfberg A. (2024). Ni-Ag Catalysts for Hydrogen Production through Dry Reforming of Methane: Characterization and Performance Evaluation. Catalysts.

[63] Mierczynski P., Kaczorowski P., Maniecki T.P., Bawolak-Olczak K., Maniukiewicz W. (2013). The influence of Pd loading on the physicochemical properties of the Cu-Cr-Al methanol synthesis catalysts. React. Kinet. Mech. Catal..

[64] Prieto P., Nistor V., Nouneh K., Oyama M., Abd-Lefdil M., Díaz R. (2012). XPS study of silver, nickel and bimetallic silver–nickel nanoparticles prepared by seed-mediated growth. Appl. Surf. Sci..

[65] X-ray Photoelectron Spectroscopy (XPS) Reference Pages. http://www.xpsfitting.com/.

[66] Biesinger M.C., Payne B.P., Grosvenor A.P., Lau L.W.M., Gerson A.R., Smart R.S.C. (2011). Resolving surface chemical states in XPS analysis of first row transition metals, oxides and hydroxides: Cr, Mn, Fe, Co and Ni. Appl. Surf. Sci..

[67] Xiao Z., Xiu J., Wang X., Zhang B., Williams C.T., Su D., Liang C. (2013). Controlled preparation and characterization of supported CuCr_2_O_4_ catalysts for hydrogenolysis of highly concentrated glycerol. Catal. Sci. Technol..

[68] Kanuri S., Dinda S., Singh S.A., Roy S., Chakraborty C., Datta S.P. (2024). Microrod networks CuO–ZnO–Al_2_O_3_ catalyst for methanol synthesis from CO_2_: Synthesis, characterization, and performance demonstration. Mater. Today Chem..

[69] Mobini S., Meshkani F., Rezaei M. (2017). Surfactant-assisted hydrothermal synthesis of CuCr_2_O_4_ spinel catalyst and its application in CO oxidation process. J. Environ. Chem. Eng..

[70] Babu G.S., Rekha V., Francis S., Lingaiah N. (2019). Vapour Phase Selective Hydrogenation of Furfural to Furfuryl Alcohol Over Cu–Cr–Zn Mixed Oxide Catalysts Prepared by Utilizing Gamma Radiation. Catal. Lett..

[71] Wang X., Sun Y., Han F., Zhao Y. (2022). Effect of Fe addition on the structure and SCR reactivity of one-pot synthesized Cu-SSZ-13. J. Environ. Chem. Eng..

[72] Ma L., Zhang Y.X., Gao X.H., Ma Q.X., Zhang J.L., Zhao T.S. (2020). Preparation of Fe_3_O_4_@PI and its catalytic performances in Fischer-Tropsch synthesis. Ranliao Huaxue Xuebao/J. Fuel Chem. Technol..

[73] Jermwongratanachai T., Jacobs G., Shafer W.D., Pendyala V.R.R., Ma W., Gnanamani M.K., Hopps S., Thomas G.A., Kitiyanan B., Khalid S. (2014). Fischer-Tropsch synthesis: TPR and XANES analysis of the impact of simulated regeneration cycles on the reducibility of Co/alumina catalysts with different promoters (Pt, Ru, Re, Ag, Au, Rh, Ir). Catal. Today.

[74] Venugopal A., Scurrell M.S. (2004). Low temperature reductive pretreatment of Au/Fe_2_O_3_ catalysts, TPR/TPO studies and behaviour in the water-gas shift reaction. Appl. Catal. A Gen..

[75] Benrabaa R., Löfberg A., Rubbens A., Bordes-Richard E., Vannier R.N., Barama A. (2013). Structure, reactivity and catalytic properties of nanoparticles of nickel ferrite in the dry reforming of methane. Catal. Today.

[76] Benrabaa R., Boukhlouf H., Löfberg A., Rubbens A., Vannier R.N., Bordes-Richard A., Barama A. (2012). Nickel ferrite spinel as catalyst precursor in the dry reforming of methane: Synthesis, characterization and catalytic properties. J. Nat. Gas Chem..

[77] Benrabaa R., Trentesaux M., Roussel P., Rubbens A., Vannier R.-N., Löfberg A. (2023). NiAlxFe_2_− xO_4_ mixed oxide catalysts for methane reforming with CO_2_: Effect of Al vs Fe contents and precursor salts. J. CO2 Util..

[78] Hallassi M., Benrabaa R., Cherif N.F., Lerari D., Chebout R., Bachari K., Rubbens A., Roussel P., Vannier R.N., Trentesaux M. (2022). Characterization and Syngas Production at Low Temperature via Dry Reforming of Methane over Ni-M (M = Fe, Cr) Catalysts Tailored from LDH Structure. Catalysts.

[79] Lee M., Oh S., Suh K., Kim D. (2002). Preparation of silver nanoparticles in hexagonal phase formed by nonionic Triton X-100 surfactant. Colloids Surf. A Physicochem. Eng. Asp..

[80] Ayyub P. (2014). Observation of a hexagonal (4H) phase in nanocrystalline silver. Phys. Rev. B.

[81] Chakraborty I., Shirodkar S.N., Gohil S. (2014). The nature of the structural phase transition from the hexagonal (4H) phase to the cubic (3C) phase of silver. J. Phys. Condens. Matter.

